# Pulmonary Vascular Resistance to Predict Right Heart Failure in Patients Undergoing Left Ventricular Assist Device Implantation

**DOI:** 10.3390/jcm13020462

**Published:** 2024-01-14

**Authors:** René Schramm, Johannes Kirchner, Mohamad Ibrahim, Sebastian V. Rojas, Michiel Morshuis, Volker Rudolph, Jan F. Gummert, Henrik Fox

**Affiliations:** 1 Clinic for Thoracic and Cardiovascular Surgery, Herz- und Diabeteszentrum NRW, Ruhr-Universität Bochum, Georgstraße 11, 32545 Bad Oeynhausen, Germany; rschramm@hdz-nrw.de (R.S.); mohamad.ibrahim@diakonie-sw.de (M.I.); srojashernandez@hdz-nrw.de (S.V.R.); mmorshuis@hdz-nrw.de (M.M.); jgummert@hdz-nrw.de (J.F.G.); 2 Clinic for General and Interventional Cardiology/Angiology, Herz- und Diabeteszentrum NRW, Ruhr-Universität Bochum, Georgstraße 11, 32545 Bad Oeynhausen, Germany; jkirchner@hdz-nrw.de (J.K.); vrudolph@hdz-nrw.de (V.R.)

**Keywords:** heart failure, pulmonary vascular resistance, left ventricular assist device, survival

## Abstract

Right heart failure (RHF) is associated with poor outcomes, especially in patients undergoing left ventricular assist device (LVAD) implantation. The aim of this study was to identify predictors of RHF after LVAD implantation. Of 129 consecutive patients (mean age 56 ± 11 years, 89% male) undergoing LVAD implantation, 34 developed RHF. Compared to patients without RHF, those with RHF required longer invasive mechanical ventilation and had longer intensive care unit and hospital stays (*p* < 0.01). One-year all-cause mortality was significantly higher in patients with versus without RHF after LVAD implantation (29.4% vs. 1.2%; hazard ratio 35.4; 95% confidence interval 4.5–277; *p* < 0.001). Mortality was highest in patients with delayed RHF after initial LVAD-only implantation (66.7%). Patients who did versus did not develop RHF had significantly higher baseline pulmonary vascular resistance (PVR; 404 ± 375 vs. 234 ± 162 dyn/s/cm^5^; *p* = 0.01). PVR > 250 dyn/s/cm^5^ was a significant predictor of survival in patients with RHF after LVAD implantation. These data confirm the negative impact of RHF on morbidity and mortality after LVAD implantation. Preoperative PVR > 250 dyn/s/cm^5^ determined using invasive right heart catheterization was an independent predictor of developing RHF after LVAD implantation, and of subsequent mortality, and could be used for risk stratification in the setting for deciding between single or biventricular support strategy.

## 1. Introduction

The prevalence of heart failure (HF) is increasing globally due to a number of factors, including the aging population demographic and more effective treatments for ischemic heart disease [1]. In addition, therapeutic advances mean that individuals with HF now live longer [1,2]. However, end-stage HF remains a therapeutic challenge, often requiring mechanical circulatory support [3]. Implantation of a left ventricular assist device (LVAD) is the most established approach for permanent mechanical circulatory support, and the use of an LVAD has been shown to be safe and effective, including for a bridge to heart transplantation [3].

Despite this, right HF (RHF) remains a challenge because available therapeutic options are limited, especially in patients ineligible for heart transplantation [3]. RHF is a known driver of both morbidity and mortality, in particular for patients who have an LVAD [4,5]. RHF can sometimes be improved by LVAD implantation because it not only unloads the left ventricle but also relieves pulmonary congestion. However, RHF persists after LVAD implantation in a significant proportion of individuals, and there is a lack of validated predictors of RHF development or resolution after LVAD implantation [3].

It has been suggested that poor right ventricular function might predict postoperative RHF after LVAD implantation [6]. Currently, the most widely used parameters for evaluating postoperative RHF are a qualitative assessment of the right ventricular size and a visual function using two-dimensional transthoracic echocardiography (2DE) [6,7]. However, there is significant inter-observer variability in 2DE measurements, and 2DE does not provide information about the complexities of right heart anatomy and the underlying disease impact [8]. Three-dimensional transthoracic echocardiography is not widely available and also often lacks sufficient image quality for detailed three-dimensional and performance measurements in the right heart [8]. Cardiac computed tomography (CT) and cardiac magnetic resonance imaging (CMR) provide high-definition three-dimensional images of the right heart, but in particular, CT has some limitations regarding functional analysis because images are conducted in a single cardiac cycle meaning that quantities such as cardiac volume and performance parameters may be misinterpreted. Another limitation of CT imaging is poor reproducibility between cardiac cycles [9,10,11]. Furthermore, right ventricular geometry is highly dependent on preload conditions [12], as well as cardiac rhythm, heart rate, blood pressure and numerous other influencing factors.

Right heart catheterization (RHC) is an established investigation to assess cardiac output and it provides detailed assessment of circulatory components. In this context, pulmonary vascular resistance (PVR) is a parameter of particular interest because it has been shown to be a predictor of outcomes in various cardiac diseases (calculation formula: 80 x (mean pulmonary arterial pressure—mean pulmonary artery wedge pressure) / cardiac output) [13]. PVR reflects right heart strain derived from vascular resistance, representing a measure of right ventricular workload. PVR is based on the hydraulic version of Ohm’s law [14], and PVR calculations require pulmonary artery pressure, pulmonary post capillary wedge pressure and cardiac output [14]. In this setting, PVR is not only a RHF parameter but also incorporates pressure-dependent aspects of the pulmonary circulation.

PVR measurements are established in the context of pulmonary hypertension [15], and studies have shown that high PVR is associated with unfavorable right ventricular remodeling and outcome in patients with tricuspid valve regurgitation [16,17]. As a marker of right heart function and congestion [18], PVR has been used as an endpoint in numerous clinical trials [19,20].

Little is known about PVR and RHF in the setting of LVAD, and published RHF risk scores, such as the Michigan risk score, Fitzpatrick risk score or European Registry for Patients with Mechanical Circulatory Support risk score, do not include PVR in their calculations and have failed to predict RHF in LVAD [21,22,23,24,25].

The aim of this study was to investigate invasive measures of RHC in patients undergoing LVAD implantation to identify predictors of RHF after LVAD implantation and their impact on survival.

## 2. Methods

### 2.1. Study Design and Population

This single-center, retrospective study included consecutive patients who underwent de novo LVAD implantation with Medtronic Heart Ware and Abbott Heart Mate3 devices, with and without a right ventricular assist device (RVAD), between 2011 and 2020. The study was approved by the institutional ethics committee.

### 2.2. Assessments

All patients were examined with transthoracic echocardiography prior to LVAD implantation and at regular intervals after LVAD implantation following current European Association of Cardiovascular Imaging (EACVI) guideline recommendations [26]. RHC was performed one week before and after LVAD implantation in all patients via Swan-Ganz catheterization using thermodilution measurement. Parameters recorded included pulmonary artery pressures, pulmonary capillary wedge pressure, right atrial and right ventricular pressures, systemic vascular resistance, PVR, and pulmonary artery oxygen saturation, plus cardiac output and cardiac index in thermodilution following the European Society of Cardiology recommendations. Clinical data collected included inotropic support, total intensive care unit (ICU) stay, total in-hospital stay, invasive mechanical ventilation and survival.

### 2.3. Right Heart Failure

RHF was defined as per the 2021 European Society of Cardiology heart failure guidelines [27]. Preoperative hemodynamics were classified using the Interagency Registry for Mechanically Assisted Circulatory Support (INTERMACS) definition (Adverse event definitions: adult and pediatric patients 2013). No standardized thresholds for RVAD indication are available. Therefore, the indication for RVAD implantation was determined on a case-by-case basis by cardiac surgeons in close consultation with the HF cardiologist and intensivist, considering hemodynamic parameters and echocardiographic measurements.

### 2.4. Endpoints

Study endpoints included survival, clinical parameters and time to hospital discharge and were compared between patients with or without RHF.

### 2.5. Statistical Analysis

Statistical analysis was performed using SPSS Statistics 27 provided by IBM (Armonk, NY, USA). Continuous and normally distributed variables are expressed as mean ± standard deviation. Receiver operating characteristic (ROC) curves were used to determine cut-off values for outcome analysis. Kaplan–Meier analysis was performed for clinical outcomes using time to first event. The study population was divided into three subgroups: one included individuals with LVAD only who developed RHF postoperatively and required secondary RVAD support, another included LVAD patients without RHF, and the third included LVAD patients with RHF who received intraoperative immediate RVAD support. Subgroups were also defined based on PVR (≥250 dyn/s/cm^5^ and <250 dyn/s/cm^5^). Differences in time-to-event distributions were evaluated by means of the log-rank test. Hazard ratio (HR) values were calculated using Cox regression. Univariate analyses were performed first and all parameters with *p* < 0.1 were then included in the multivariate analysis. A *p*-value of <0.05 was considered statistically significant.

## 3. Results

### 3.1. Study Population

A total of 129 patients were included (mean age 56 years, 89% male) (Table 1). Nearly three-quarters of participants had isolated left HF and received an LVAD only, and thirty-four also had RHF (biventricular HF) (Table 1). Of those with RHF, 28 had immediate temporary RVAD implantation intraoperatively, and 6 developed RHF postoperatively and required secondary RVAD support after initial LVAD-only implantation. Mean follow-up was 222 ± 59 days.

### 3.2. Left Ventricular Function and Invasive Hemodynamics

All transthoracic echocardiography values were indicative of severely impaired cardiac function (Table 2). Mean SVR and PVR were significantly higher in individuals with both right and left HF compared to those with left-sided HF only (Table 2). In the LVAD group, 66/95 patients (68%) required inotropic therapy, most often milrinone or dobutamine, while 28/34 (82%) of those with both left and right HF needed inotropes, also usually milrinone or dobutamine; requirement for inotropic therapy did not differ statistically significantly between groups, with numerically more dobutamine support in the LVAD-only group (Table 3).

### 3.3. Outcomes

**Figure 1:**
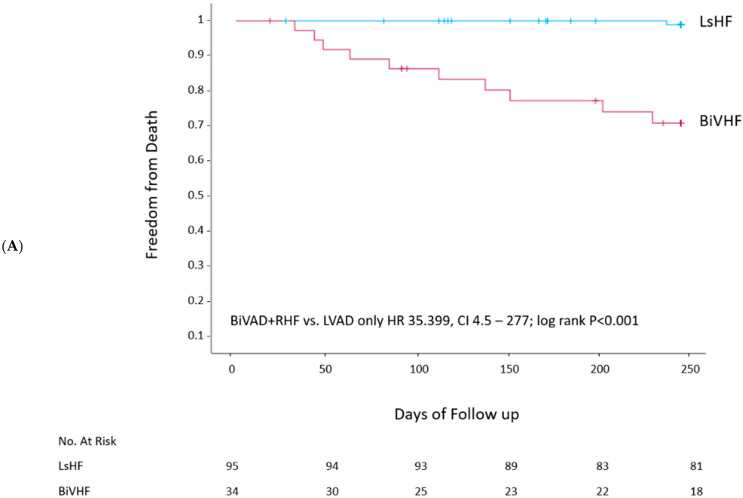

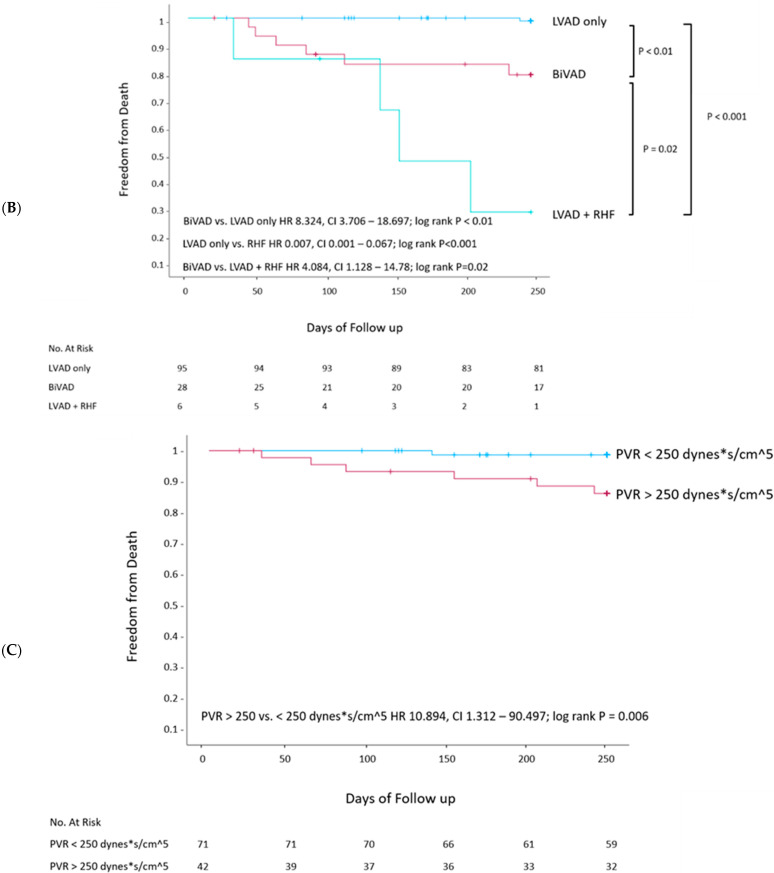
Kaplan–Meier survival curves. (**A**) Patients with left heart failure (LsHF) or left and right (bilateral ventricular) heart failure (BiVHF); (**B**) left ventricular assist device (LVAD) implantation, simultaneous LVAD and right ventricular assist device implantation (BiVAD); (**C**) LVAD implantation with subsequent development of right heart failure (LVAD + RHF). CI, confidence interval; HR, hazard ratio.

Death occurred in 11/129 (8.5%) patients overall, 1 (1.1%) in the LVAD-only group and 10 (29.4%) in the group that also had RHF (HR 35.4; 95% confidence interval [CI] 4.5–277; log rank *p* < 0.001) (Table 4, Figure 1A). The duration of mechanical ventilation and time spent in the ICU were significantly longer in the RHF versus LVAD-only group (Table 4). LVEF did not change significantly from baseline in either group (Table 4).

### 3.4. Subgroups and Predictors

Individuals who developed RHF postoperatively and required secondary RVAD support had the worst overall survival (HR LVAD only vs. secondary RVAD 18.96; 95% CI 5.36–66.99; *p* < 0.01; and HR LVAD only vs. LVAD with intraoperative RVAD 4.41; 95% CI 1.13–14.78; *p* = 0.02). Survival was best in the group that had LVAD but did not develop RHF and intermediate in those who had LVAD implantation with RHF and intraoperative immediate RVAD support (HR LVAD without RHF vs. LVAD only 8.3; 95% CI 3.71–18.70; *p* < 0.001) (Figure 1B). The device manufacturer (HeartWare versus HeartMate3) did not influence the results or RHF occurrence in the sensitivity analysis.

Pre-LVAD implantation PVR > 250 dyn/s/cm^5^ was the only significant independent predictor of mortality on multivariate Cox regression analysis (HR [95% CI] value for risk of death in individuals with a preimplantation PVR of >250 vs. <250 dyn/s/cm^5^ 10.89 [1.31–90.50]; log rank *p* = 0.006) (Table 5).

## 4. Discussion

This is the first and largest study to use RHC to evaluate the impact of RHF in individuals undergoing LVAD implantation. We identified preoperative PVR > 250 dyn/s/cm^5^ as being associated with the development of RHF and as a significant independent predictor of survival, after LVAD implantation. Patients who developed RHF after LVAD implantation required significantly more hours of invasive mechanical ventilation, spent longer in the ICU and in hospital, and had the worst survival compared with patients who did not develop RHF or had a RVAD implanted at the same time as an LVAD. This is clinically relevant because there is currently no satisfactory therapy for RHF in patients on an LVAD.

RHF has previously been shown to be associated with a higher risk of mortality after LVAD implantation [28]. However, available studies have not reported consistent results and used differing definitions of RHF. In general, RHF is diagnosed when patients undergoing LVAD require mechanical right ventricular support, show elevated vena cava pressures, show signs of liver congestion and/or ascites, or require continuous inotropic support for ≥14 days. However, all of these parameters represent postoperative clinical findings that cannot provide a preoperative indication of the risk of developing postoperative RHF, which would be useful to know because patients developing RHF in the postoperative course and requiring secondary subsequent RVAD support had by far the worst outcomes in our study. However, excessive or prophylactic RVAD supply during LVAD implantation prolongs the surgical procedure and carries additional risks, such as infection, bleeding, vessel injury and wound healing complications. Therefore, RVAD supply should only be used in patients undergoing LVAD who definitely need right ventricular support. However, there is currently no information about how to determine the requirement for RVAD during LVAD implantation, so our study adds important information to the field based on RHC findings.

Several calculation scores for RHF prediction after LVAD implantation have been developed over recent years. One of these is the Fitzpatrick score, which combines clinical parameters (e.g., previous cardiac surgery) with laboratory findings (e.g., creatinine) [21]. The Fitzpatrick score also included echocardiographic parameters indicating right ventricular dysfunction and the need for treatments such as inhaled nitric oxide or intravenous catecholamines. Similar parameters are included in the EUROMACS score [25]. However, all currently available scores have failed to reliably predict the postoperative development of RHF after LVAD implantation.

It was previously unclear whether immediate or belated RVAD support implantation had the ability to reduce mortality in patients at risk of RHF after LVAD implantation. This is relevant because observational studies have reported that RVAD support within the first 6–12 months after LVAD implantation is associated with significantly higher rates of bleeding, infection and death [29,30]. Therefore, to reduce the occurrence of these adverse events, interdisciplinary heart team decisions tend to favor LVAD-only strategies, even in patients with suspected RHF, with the potential for improvement in right ventricular function after LVAD implantation. However, there are not any currently validated predictors of right ventricular function recovery after LVAD implantation, due at least in part to the fact that studies have used heterogenous strategies even within LVAD centers and differing patient pathways [31,32].

The validity of the LVAD-only approach was confirmed by our data because this group had the best outcome at 250 days after LVAD implantation. However, participants who had immediate RVAD support implantation had much better outcomes than those who had later RVAD implantation, suggesting a beneficial impact of early RVAD support in patients with RHF. Given the lack of clear definitions relating to the need for RVAD, decisions about RVAD implantation in our study were made on a case-by-case basis. The risks of simultaneous RVAD and LVAD implantation but the better outcomes seen with this approach, as compared to an initial LVAD-only strategy followed by secondary RVAD addition, in patients with right ventricular heart failure who really need right ventricular support, means that there is a need for preoperative parameters that can be used to identify the best candidates for the addition of an RVAD during LVAD implantation.

Although the novelty of this study is an important strength, some limitations need to be considered when interpreting the findings of this study. First, it had a retrospective, single-center design, and decisions about RVAD implantation were made on a case-by-case basis by an interdisciplinary HF board rather than based on standardized criteria (which were not available during the study period). Second, we prospectively included de novo LVAD implantations, but device exchange procedures and revisions were excluded. This could have introduced a selection bias and means that the findings are only generalizable to de novo LVAD implantation procedures. Third, it is important to be aware that the findings can only be generalized to, and compared with, situations using the same definition of RHF. Moreover, only centrifugal LVADs have been used and allow conclusions from our findings, and the group of delayed right heart failure patients is rather small, allowing limited conclusions only. Finally, most variables in the limited set of parameters evaluated in this study are dynamic and might change over time based on volume management strategies.

## 5. Conclusions

This study makes an important contribution to the unresolved topic of RHF on an LVAD. The results showed a significant increase in the mortality risk in patients with RHF and LVAD, highlighting the need for parameters that identify which patients in this setting need early RVAD support. Our data showed that a preoperative PVR dyn/s/cm^5^ could be a useful marker of the need for right ventricular support. This now needs to be investigated in prospective, controlled trials because RHF remains a significant challenge in all patients undergoing LVAD implantation.

## Figures and Tables

**Table 1:** Baseline characteristics (at the time for decision for LVAD) of the study population.

Characteristics	Total (*n* = 129)	LVAD Only (*n* = 95)	LVAD + RHF (*n* = 34)	*p*-Value
Age, years	56 ± 11	57 ± 10	54 ± 13	0.194
Male sex, *n* (%)	116 (89%)	84 (88%)	32 (94%)	0.512
Urea, mg/dL	70 ± 43	69 ± 42.6	71.8 ± 46	0.794
Creatinine, mg/dL	1.6 ± 1	1.5 ± 1	1.7 ± 1	0.845
Bilirubin, mg/dL	1.5 ± 1.6	1.4 ± 1.2	2 ± 2.5	0.196
ALT, U/L	70 ± 158	71 ± 179	65 ± 79	0.422
AST, U/L	51 ± 105	53 ± 120	47 ± 40	0.784
CRP, mg/dL	3.4 ± 4.9	3.0 ± 4.4	4.4 ± 6	0.154

Values are mean ± standard deviation or number of participants (%). CRP, C-reactive protein; LVAD, left ventricular assist device; RHF, right heart failure; ALT, alanine aminotransferase; AST, aspartate aminotransferase.

**Table 2:** Transthoracic echocardiography data for left ventricular function and hemodynamics at baseline.

Parameter	Total (*n* = 129)	LVAD Only (*n* = 95)	LVAD + RHF (*n* = 34)	*p*-Value
LVEF, %	21.5 ± 6.2	21.5 ± 6.2	21.5 ± 6.5	0.961
LVEDD, mm	72 ± 11	71 ± 11	73 ± 13	0.51
LVESD, mm	66 ± 12	66 ± 11	65 ± 15	0.732
Heart rate, beats/min	87 ± 18	86 ± 17	89 ± 20	0.414
Mean PAP, mmHg	33 ± 11	32 ± 7	33 ± 9	0.983
PCWP, mmHg	22 ± 10	22 ± 11	22 ± 7	0.94
CO, L/min	5.1 ± 7.9	4.7 ± 4.9	6.2 ± 13.3	0.339
CI, L/min/m^2^	2.1 ± 0.64	2.1 ± 0.67	2.02 ± 0.59	0.515
SVR, dyn/s/cm^5^	4152 ± 1483	1354 ± 620	1904 ± 942	0.008
PVR, dyn/s/cm^5^	269 ± 231	234 ± 162	404 ± 375	0.01

Values are mean ± standard deviation. CI, cardiac index; CO, cardiac output; LVAD, left ventricular assist device; LVEDD, left ventricular end-diastolic diameter; LVEF, left ventricular ejection fraction; LVESD, left ventricular end-systolic diameter; PAP, pulmonary artery pressure; PCWP, pulmonary capillary wedge pressure; PVR, peripheral vascular resistance; RHF, right heart failure; SVR, systemic vascular resistance.

**Table 3:** Inotropic therapy.

Inotropes	Total (n = 129)	LVAD Only (n = 95)	LVAD + RHF (n = 34)	*p*-Value
Milrinone	67 (52%)	49 (52%)	18 (53%)	0.99
Dobutamine	64 (50%)	49 (52%)	15 (44%)	0.55
Levosimendan	2 (2%)	2 (2%)	0 (0%)	0.99
Epinephrine	2 (2%)	1 (1%)	1 (3%)	0.459
Dopamine	14 (11%)	5 (5%)	9 (26%)	0.002
Norepinephrine	3 (2%)	3 (3%)	0 (0%)	0.566

Values are number of patients. LVAD, left ventricular assist device; RHF, right heart failure.

**Table 4:** Outcomes and follow-up.

	Total (*n* = 129)	LVAD Only (*n* = 95)	LVAD + RHF (*n* = 34)	*p*-Value
Death, n (%)	11 (8.5)	1 (1.1)	10 (29.4)	<0.01
Duration of invasive mechanical ventilation, days	227 ± 535	101 ± 200	602 ± 922	<0.001
Duration of ICU stay, days	28.4 ± 39.5	15.5 ± 19.7	64.4 ± 56	<0.001
LVEF, %	24.6 ± 8	23.9 ± 6.5	26.8 ± 11	0.068
LVEDD, mm	59 ± 13	59 ± 13	58 ± 13	0.516
LVESD, mm	55 ± 13	55 ± 13	54 ± 14	0.729
Urea, mg/dL	52 ± 33	49 ± 31	61 ± 38	0.094
Creatinine, mg/dL	1.3 ± 1.1	1.3 1.7	1.4 ± 0.8	0.697
Bilirubin, mg/dL	1 ± 1.9	0.68 ± 0.25	1.9 ± 0.25	0.06
ALT, U/L	24 ± 26	19 ± 13	37 ± 42	0.045
AST, U/L	33 ± 34	27 ± 10	47 ± 63	0.104
CRP, mg/dL	4.1 ± 4.2	3.4 ± 3.1	5.9 ± 6.1	0.96

Values are mean ± standard deviation or number of participants (%). CRP, C-reactive protein; LVAD, left ventricular assist device; LVEDD, left ventricular end-diastolic diameter; LVEF, left ventricular ejection fraction; LVESD, left ventricular end-systolic diameter; RHF, right heart failure; ALT, alanine aminotransferase; AST, aspartate aminotransferase.

**Table 5:** Predictors of survival after left ventricular assist device implantation.

	Univariate Cox Regression		Multivariate Cox Regression	
	HR (95% CI)	*p*-Value	HR (95% CI)	*p*-Value
Age	1.00 (0.95–1.06)	0.916	-	
Sex	0.88 (0.11–6.90)	0.905	-	
LVEF, %	1.09 (0.99–1.20)	0.079	1.07 (0.95–1.19)	0.272
Bilirubin, mg/dL	1.21 (1.04–1.40)	0.012	0.53 (1.57–1.79)	0.307
Urea, mg/dL	1.00 (0.98–1.01)	0.775	-	
ALT, U/L	1.00 (0.99–1.01)	0.914	-	
AST, U/L	0.99 (0.98–1.01)	0.437	-	
Creatinine, mg/dL	1.20 (0.77–1.86)	0.419	-	
Mean PAP, mmHg	1.01 (0.95–1.06)	0.817	-	
PVR >250 vs. <250 dyn/s/cm^5^	10.89 (1.31–90.50)	0.027	10.38 (1.21–89.04)	0.033
SVR, dyn/s/cm^5^	1 (1.00–1.00)	0.773	-	
PCWP, mmHg	0.99 (0.93–1.07)	0.867	-	
Cardiac index	0.93 (0.37–2.33)	0.872	-	

CI, confidence interval; HR, hazard ratio; LVEF, left ventricular ejection fraction; PAP, pulmonary artery pressure; PCWP, pulmonary capillary wedge pressure; PVR, peripheral vascular resistance; ALT, alanine aminotransferase; ALT, aspartate aminotransferase.

## Data Availability

All data included in this manuscript.

## Abbreviations

2DE	two-dimensional transthoracic echocardiography
CI	confidence interval
CT	computed tomography
EACVI	European Association of Cardiovascular Imaging
HF	heart failure
HR	hazard ratio
LVAD	left ventricular assist device
LVEF	left ventricular ejection fraction
PVR	pulmonary vascular resistance
RHC	right heart catheterization
RHF	right heart failure
ROC	receiver operating characteristic
RVAD	right ventricular assist device
